# Spanish Adaptation and Validation of the Outcome Questionnaire OQ-30.2

**DOI:** 10.3389/fpsyg.2017.00673

**Published:** 2017-05-16

**Authors:** Paula Errázuriz, Sebastián Opazo, Alex Behn, Oscar Silva, Sergio Gloger

**Affiliations:** ^1^Department of Psychology, Pontificia Universidad Católica de ChileSantiago, Chile; ^2^National Research Center for Integrated Natural Disaster ManagementSantiago, Chile; ^3^Millennium Institute for Research in Depression and PersonalitySantiago, Chile; ^4^Psicomédica Clinical and Research GroupSantiago, Chile; ^5^Department of Psychiatry, Universidad de ChileSantiago, Chile

**Keywords:** adaptation, validation, psychotherapy outcome, measurement, psychotherapeutic process

## Abstract

This study assessed the psychometric properties of a Spanish version of the Shortened Outcome Questionnaire (OQ-30.2, Lambert et al., 2004) validated with a sample of 546 patients in an outpatient mental health clinic and 100 non-clinical adults in Chile. Our results show that this measure has similar normative data to the original measure, with a cutoff score for the Chilean population set at 43.36, and the reliable change index at 14. This Spanish OQ-30.2 has good internal consistency (α = 0.90), has concurrent validity with the Depressive, Anxious, and Somatoform disorders measuring scale (Alvarado and Vera, 1991), and is sensitive to change during psychotherapy. Consistent with previous studies, factorial analyses showed that both, the one-factor solution for a general scale and the three-factor solution containing three theoretical scales yielded poor fit estimates. Overall, our results are similar to past research on the OQ-45 and the OQ-30. The short version has adequate psychometric properties, comparable to those of the OQ-45, but provides a gain in application time that could be relevant in the setting of psychotherapy research with large samples, frequent assessments over time, and/or samples that may require more assistance completing items (e.g., low-literacy). We conclude that this measure will be a valuable instrument for research and clinical practice.

## Introduction

The Outcome Questionnaire OQ-30.2, developed by Lambert et al. (2004), measures progress in psychological functioning during treatment, in the context of both private and public health care. This measure is a shortened version of the OQ-45.2 (Outcome Questionnaire; Lambert et al., 1996), which monitors progress on three dimensions: subjective discomfort, interpersonal relationships, and social role performance (Lambert, 1983). These dimensions are intended to monitor an overall performance of the patient, but are not intended as a diagnostic tool. The OQ-30.2 was designed to be sensitive to change over short periods of time, and to assess common symptoms across a wide range of mental disorders in adults. Patients are requested to answer the questionnaire several times during treatment, and their performance is to be contrasted with both the performance of the general population, and their own performance over time.

The item selection for the OQ-30.2 was determined by prioritizing items that addressed common problems in the population and assessed social characteristics related to quality of life (Lambert et al., 2004). While being 15 items shorter than the OQ-45.2, and thus faster to administer, it has been claimed that the OQ-30.2 maintains the psychometric properties of validity, reliability, and sensitivity to change.

Studies on the OQ-45 have reported high internal consistency (0.93) and test–retest reliability (0.84), and high correlations with a set of outcome measures, such as Beck’s Depression Inventory, the Symptom Checklist 90R, and Taylor’s Manifest Anxiety Scale (Burlingame et al., 1995). As argued by Vermeersch et al. (2000), a measure has good sensitivity to change if it captures patient change in the hypothesized direction (in the case of the Outcome Questionnaire, a negative slope) in the setting of an intervention that is thought to mobilize change. Additionally, a measure is thought to be sensitive to change when the rate of change is significantly more pronounced in treated individuals versus untreated individuals. The OQ-45 has shown to be sensitive to change with a large effect size (*d* = 0.59), detecting improvements in clients receiving psychotherapy and not in untreated individuals (Vermeersch et al., 2000, 2004).

Ever since the construction of the measure, the OQ-45 has been widely used in clinical settings, across cultures. Similar psychometrics have been found in studies conducted in Italy (Chiappelli et al., 2008), Israel (Gross et al., 2015), Germany (Lambert et al., 2002), Sweden (Wennberg et al., 2010), Netherlands (de Jong et al., 2007), Portugal (Machado and Fassnacht, 2015), and Chile (Von Bergen and De la Parra, 2002).

Similar to its predecessor, studies on the OQ-30.2 have also shown high internal consistency (0.93) and test–retest reliability (0.84), and high correlation coefficients with frequently used outcome scales (Lambert et al., 2004). Additionally, it is important to note that 27 of the 30 items used in the OQ-30.2 were reported as highly sensitive to change (Vermeersch et al., 2000, 2004). Compared to the OQ-45, the shorter OQ-30.2 has not been used as widely and thus, studies focusing on its psychometric properties are not as ubiquitous (Burchett et al., 2016). Most studies involving the OQ-30.2 have focused on either treatment effectiveness (Minami et al., 2009) or feedback systems (Brown and Jones, 2005). Moreover, it is even harder to find cross-cultural validation studies of the OQ-30.2.

In one study, Ellsworth et al. (2006) analyzed the level of agreement between the OQ-45 and the OQ-30 in measuring treatment progress. According to their results, both measures showed excellent agreement in classifying clients as being functional or dysfunctional, and were very similar in classifying clients as being recovered, improved, not changed or deteriorated (Ellsworth et al., 2006).

### Measuring Psychotherapy Outcomes in Chile

Regarding the factorial structure of the instrument, there have been multiple attempts to replicate the theoretical factor solution (three scales, three factors) or explore alternatives structures for both the OQ-45 and the OQ-30 (Lo Coco et al., 2008; Minami et al., 2009; Bludworth et al., 2010; Kim et al., 2010). Lambert et al. (2004) themselves argue that both a “one factor” solution for the OQ-30 as well as a “three factor” solution for the OQ-30 have a good fit, but because the three subscales are highly correlated a one-factor solution for symptomatic distress has been advanced by Lambert et al. (2004).

In the Chilean context, there are insufficient tools to assess psychotherapeutic process because very few measures have been validated. An exception is the Spanish adaptation and validation of the OQ-45.2 (Von Bergen and De la Parra, 2002) based on a sample of 253 adults (110 men and 143 women), ages 16 to 58, of which 124 were psychiatric patients and 129 adults who reported no need for mental health treatment. The Spanish OQ-45.2 showed high internal consistency (α = 0.91) and high test–retest reliability (*r* = 0.90) with a period of 2 weeks between the first and second application. The results correlated positively with depressive, anxious, and somatoform symptoms measured with previously validated questionnaires for the Chilean population. An acceptable sensitivity to change was found, where scores were significantly lower post-test (*M* = 76.6 *SD* = 24.2) than pre-test (*M* = 96.3 *SD* = 20.4), *t* = 6.19, *p* < 0.01 in the clinical sample.

Since its validation in Chile, the OQ-45 has been widely used in local research with diverse clinical and non-clinical populations. For example, while one study used the OQ-45 to measure psychopathology in a psychiatric hospitalization unit (Correa et al., 2006), another study measured psychological functioning in a group of depressed women in a primary care setting (Ballesteros et al., 2007). Yet, a third study used the OQ-45 with a non-clinical population to measure change in stress levels in healthcare workers after participating in a mindfulness stress reduction program (Medeiros and Pulido, 2011). It is important to note that most psychotherapy studies that have used the OQ-45 in Chile report results on the general scale rather than on the subscales.

Given that the literature shows that the shorter version has comparable psychometric properties than its longer predecessor (Jones, 2004), the gain in application time and the relative underuse of subscale data were estimated to be sufficient conditions to (a) validate the OQ-30.2 in a Chilean sample, and (b) to further explore ongoing issues of the OQ, including its factorial structure. This will allow for the systematic monitoring of psychological functioning, and psychotherapeutic progress in a simpler, shorter, and less expensive manner, since it will take only two thirds of the time to administer the measure, and it will also speed up the scoring process. This is especially relevant in a country like Chile, where up to 53% of adults have low level of literacy and may require assistance completing measures such as the OQ-30.2 (Organization for Economic Co-operation Development [OECD], 2016). In addition, a shorter questionnaire is less anxiety-provoking, making it more likely that patients in research and clinical settings will agree to answer it. Thus, the current study presents the Spanish adaptation and psychometric properties (reliability, convergent validity, change sensibility, and factorial structure) of the OQ-30.2 in Chile, using a clinical and a non-clinical sample.

We hypothesized that the OQ-30.2 validated with a Chilean sample would have similar psychometric properties to the original OQ-30.2 (Lambert et al., 2004). More specifically, we expected this measure to have similar normative data, cutoff score, and reliable change index (RCI) to the original measure. We also hypothesized that this measure would be reliable, would have concurrent validity with the Depressive, Anxious, and Somatoform disorders measuring scale (DAS; Alvarado and Vera, 1991), and would be sensitive to change during psychotherapy. Finally, based on previous research (Kim et al., 2010), we expected that the theoretical three-factor structure would not be replicated.

## Materials and Methods

### Participants

Data was collected in Santiago Chile from a non-clinical sample and an outpatient clinical sample in order to establish the normative characteristics of the instrument. **Table 1** shows the demographic characteristics of both samples. Independent-sample *t*-tests were conducted to compare age (*Contrast* = -1.88, *SD* = 1.83, *t* = -1.03, *p* = 0.88, 95% CI [-5.48, 1.73]), gender (*Contrast* = 0.003, *SD* = 0.05, *t* = 0.06, *p* = 0.64, 95% CI [-0.09, 0.10]), and SES (*Contrast* = -0.19, *SD* = 0.27, *t* = -0.72, *p* = 0.44, 95% CI [-0.72, 0.33]), revealing no significant differences between non-clinical participants and outpatient participants.

**Table 1:** Demographic characteristics of participants.

Sample	*N*	Age	Gender	SES (monthly income)
Non-clinical sample	100	*M* = 40.4	Male = 24 (24%)	*M* = U$ 1,180
		*SD* = 14.6	Female = 76 (76%)	*SD* = U$ 855
Clinical sample	546	*M* = 41.3	Male = 137 (25%)	*M* = U$ 1,200
		*SD* = 12.8	Female = 409 (75%)	*SD* = U$ 735


The non-clinical sample included 100 adults surveyed outside a shopping mall in Santiago who explicitly reported not being in mental health treatment at the time, and also reported no need of mental health treatment of any kind. The average years of education was 14 (*SD* 2.5) and 13% of the participants belonged to an ethnic minority.

The clinical sample included 546 adults who began psychotherapy at a private outpatient mental health center. All patients were invited to participate and there was no exclusion criterion. Most of them (87.1%) were in psychiatric treatment at the time, 89.7% were taking psychopharmacology, and 8.8% had previously been hospitalized for mental health problems or substance abuse. Most of patients with an Axis I diagnosis were diagnosed with depressive disorders (73.5%), bipolar disorder (6.0%), adjustment disorder (1.2%), or dysthymic disorder (1.2%). In addition, 27.7% received a diagnosis of at least one comorbid Axis I disorder. The most prevalent comorbid diagnoses were substance-related disorders (4.8%), panic disorder without agoraphobia (4.8%), and dysthymic disorders (3.0%). Most patients with an Axis II diagnosis were diagnosed with dependent (2.4%), borderline (1.8%), and histrionic personality disorder (0.6%). Average years of education were 15 years (*SD* 3.0) and 5.3% of the participants belonged to an ethnic minority. The mental health disorders presented in this sample are representative of the most common mental health issues in the Chilean population (Organization for Economic Co-operation, and Development [OECD], 2013).

All therapists that took part in this study had a professional degree in psychology, graduating from a 5-year full-time professional program, which usually includes 1 or 2 years of clinical training. All patients were treated in individual therapy, independently of their reason for consulting or their therapists’ theoretical perspective. The usual treatment at this mental health center, relies on an integrative approach that uses multiple techniques from different theoretical approaches. The duration of each session was approximately 50 min.

### Instruments

#### Spanish Version of the OQ-30

The OQ-30.2 is a shorter version of the OQ-45.2. We constructed the Spanish version of the OQ-30.2 from a selection of existing items in the OQ-45.2 validated by De la Parra and Von Bergen (2002). These authors translated to Spanish the 45.2 items from the original measure, which involved simplifying vocabulary, adding explanatory notes to some items, and changing the format of the measure. Additionally, statements that included a “double negation” in Spanish were rewritten and their value changed to positive. This implies that when scoring, these items should be scored in reverse. With this as a precedent, we selected the same 30 items selected by Lambert et al. (2004) when they created the OQ-30.2 from the 45 items validated by De la Parra and Von Bergen, replicating the format of the original measure.

The administration of the measure was performed according to the parameters set in the original version of the measure (Lambert et al., 2004). The OQ-30.2 is primarily self-administered and requires no instructions beyond those printed on the answer sheet. However, according to De la Parra and Von Bergen (2002), it was necessary to express the directions orally in the first administration, emphasizing the confidentiality of responses and the meaning of the Likert scale, and answering questions that arose while the participant answered the measure. In the case of illiterate participants, the entire measure was administered orally each time.

In order to calculate the OQ-30.2 total score all items should be summed, each of which are scored on a five-point Likert scale (from 0–4), totaling a maximum score of 120 points. According to Lambert et al. (2004, p. 2), “The higher the score, the more distress the individual is acknowledging.” As in the original English version, items 5, 9, 18, and 30 should be scored in reverse. In addition, due to the translation into Spanish, items 22 and 27 also must be scored in reverse. Omitted answers should be imputed with the average value of all answered items rounded to the nearest whole number (Lambert et al., 2004).

#### DAS Scale

The DAS scale is a 35-item questionnaire that measures symptoms under three sub-scales: Anxiety, Depression and Somatoform symptoms. In 1991, Alvarado et al. validated this scale in Chile, and Von Bergen and De la Parra used it in 2002 as a concurrent validity measure. Similarly, it was used in this study as a concurrent validity measure.

#### Background Questionnaire

We developed a 13-item self-administered questionnaire to collect sociodemographic information, including gender, age, nationality, monthly family income, educational level, employment status, marital status, and religious belief. In addition, we included items about previous psychotherapy and psychiatric treatments, and the perceived need for psychological or psychiatric support at the moment. This questionnaire was constructed using as reference items included in the 2012 Chilean census, and was employed both to compare the surveyed population with the one originally utilized by Lambert, and to assure demographic equivalence between the clinical and control samples.

#### Procedure

For the clinical sample, all new psychotherapy clients were invited to participate in our study when they called to schedule their first appointment. Those who agreed to participate signed an informed consent before data was collected. Participants completed the OQ-30.2 before their first, sixth and last psychotherapy sessions and the background questionnaire before the first session. In the clinical sample the questionnaire was administered in the waiting room before a psychotherapy session. Administration time for the first session took between 5 and 10 min (including oral directions), and in the case of illiterate participants between 15 and 20 min. As expected, administration time tended to decrease significantly in subsequent sessions, likely a result of increasing familiarity with the measure.

Meanwhile, the non-clinical sample was obtained by surveying people outside a large shopping mall near the mental health clinic. Those who agreed to participate first signed a consent form and then answered once the background questionnaire, OQ-30.2, and DAS. The administration time for this one-time data collection was between 5 and 10 min as well.

The consent forms used in the clinical and non-clinical samples included detailed information about the study’s procedures, which were approved by the relevant ethical review boards.

### Data Analysis

Because the way each psychometric property is calculated is central to this paper, the details of each analyses are presented in the results section together with the actual calculations. We first conducted a Confirmatory Factor Analysis (CFA) in order to test the most appropriate factor structure of the OQ-30.2 using MPLUS 7 (Muthén and Muthén, 1998–2011). Despite the extensive clinical use of the OQ-45 and the OQ-30.2, the theoretical three-factor structure has not been empirically replicated (Kim et al., 2010). Because of this shortcoming, Lambert et al. (2004) point out that the OQ-30.2 should be scored in a single scale, representing one factor of symptom distress. However, each item originally belongs to one of the three OQ-45 subscales: “OQ Symptom Distress,” “OQ Interpersonal Relations,” and “OQ Social Role.” Candidate factor solutions were analyzed in regards to the following model fit indices: (1) Tucker-Lewis Index (TLI) also known as Non-Normed Fit Index, (2) Comparative Fit Index (CFI), and (3) Root Mean Square Error of Approximation (RMSEA). These fit indices have been proposed as reliable to analyze factor solutions using CFA (Schreiber et al., 2006). To estimate model fit using these indices we used cutoff levels proposed by Hu and Bentler (1999): (TLI > 0.95 CFI > 0.95, and RMSEA < 0.06). Additionally, a bi-factor model was tested including a general factor as well as the specific factors of the scale where the general factor is specified to be uncorrelated with specific factors and these are also specified to be uncorrelated to each other (Harman, 1976). A bi-factor model is useful to examine the pertinence of maintaining both a one-dimensional (general scale only) and a multi-dimensional (specific subscales) at the same time (Reise et al., 2010).

Second, we present normative data that resulted from calculating the mean and the standard deviation of each sample.

Third, we report the cutoff score. As has been previously established by Lambert et al. (2004), the cutoff score is the score that differentiates the mentally dysfunctional population from the population that operates within normal ranges. Hence, if a particular score is below the cutoff score, it can be considered as reporting psychological dysfunction, and vice versa. The cutoff score was calculated with the data taken from both clinical and non-clinical samples, using the criteria and formulas presented by Lambert et al. (2004), and replacing the original values with the current ones.

Fourth, we calculated the RCI, which establishes when a change between scores is statistically significant. This calculation is crucial for analyzing patients’ change during treatment, allowing the clinician to know when a clinically significant change has occurred. Like the previous section, we used the formula proposed by Lambert et al. (2004) using the internal consistency value of the OQ-30, and a pooled standard deviation value (SD).

Fifth, we calculated the reliability by using the Cronbach alpha coefficient as an expression of the internal consistency of the total scale. This coefficient is used to assess the degree of homogeneity among the items, i.e., whether the items of the same scale are evaluating a concept common to all of them. As a general rule, to consider an instrument as having good internal consistency the alpha value must be greater than or equal to 0.70 (Cohen, 1992). We expected to obtain similar values to the original OQ-30.2 scale (Non-Clinical sample α = 0.93, Clinical sample α = 0.93; Lambert et al., 2004).

Sixth, we calculated the concurrent validity for the measure using the Pearson product moment correlation between the OQ-30.2 and DAS scale. We deem it pertinent to analyze correlations between the DAS and OQ-30.2 items by grouping them in the three original OQ dimensions, as the DAS measures symptoms primarily.

Seventh and last, we estimated the internal responsiveness and change sensitivity of the OQ-30.2. Internal responsiveness was calculated using the standardized response mean effect size, also known as the Responsiveness-Treatment (RT) coefficient (Husted et al., 2000). The RT is calculated as the ratio of change scores between the first and last application of the measure (mean change score) and the standard deviation of change scores. Because the RT coefficient is a measure of the size of the effect, it can be interpreted using Cohen’s benchmarks (Cohen, 1988) where values of 0.20 or less, 0.50 and 0.80 and greater are indicative of small, moderate, and large responsiveness (Husted et al., 2000). To measure sensitivity to change we used a mixed-effects modeling framework to estimate growth parameters, in particular the slope of the OQ-30.2 trajectory over time (Tasca and Gallop, 2012). The mixed-effects model has been regarded as one of the most prominent statistical methods for analyzing longitudinal data, for its ability to handle missing data which may come from incomplete measurements, illness, death, or dropout (Xu and Blozis, 2011). In a mixed model, “an individual’s score is assumed to be due to one or more latent variables that represent individual-level characteristics of growth, such as intercept and slope” (Xu and Blozis, 2011, p. 238). In the case of capturing change, a latent process of score-improvement (or deterioration in the variable of interest) may be adequately captured by these models so that they can be a reliable tool to examine sensitivity to change when change is conceptualized to be a latent variable (Blozis, 2004; Xu and Blozis, 2011). This poses a contrast to traditional methods of assessing sensitivity to change that rely on estimates based on empirically measured outcomes (see for a review, Guyatt et al., 1987). Since growth curve modeling is essentially a multilevel linear modeling strategy it allows for the estimation of growth parameters (intercept and slope) considering within-patient change and between-patient heterogeneity in change rates. For this procedure data for the first four psychotherapy sessions was used, because it captured most of the available data. After the fourth session, there is significant dropout in cases, and thus, modeling based on a larger number of sessions can affect the precision of the model estimates in the sense that it may yield unreliable estimates of growth parameters. As was previously mentioned, a measure has good sensitivity to change if it captures patient change, in this case a negative slope. This change also needs to be significantly more pronounced in treated individuals. This second element could not be assessed in the current study, because no longitudinal data was collected for the non-clinical group. Thus, estimates of sensitivity to change are produced and interpreted in relationship to the first condition stated, that is, the property of the instrument to capture change in the hypothesized direction when change is thought to occur because of an intervention.

## Results

### Confirmatory Factor Analyses

Before running the factor analysis, multivariate normality was assessed using Mardia’s Test of Multi-normality (Mardia, 1970). The statistic was estimated using Mplus 7 (Version 1.4). Results of the test indicated that the assumption of multivariate normality was violated (Sample value: 149.632, *p* = 0.000). Thus, we used maximum likelihood with robust standard errors as an estimation technique for the factor analysis. This estimation procedure constitutes a robust technique for skewed data structures (Fuller and Hemmerle, 1966; Finney and DiStefano, 2006).

Two factor-structure solutions as well as a bi-factor analysis were tested and compared in regard to multiple fit indices proposed in the CFA literature (for a review, see Schreiber et al., 2006). **Table 2** presents a model fit for a one-factor solution, three-factor solution and a bi-factor solution. As can be seen, all three solutions yield relatively poor fit indices for the observed sample. For all three solutions, the value for the CFI and TLI are below the desired 0.95 threshold, and the RMSEA indices are above the 0.06 threshold. The three-factor solution provides a marginally better fit, but it falls under acceptable levels. **Table 3** presents specifics factor loadings for the theoretical scales of the instrument given a three-factor structure. It can be observed that item selection for the OQ-30.2 was efficient in that almost all items load significantly on the factors in both, the three-factor and the one-factor solution. In the three-factor solution, only item number 24 -which maps on social role problems related to problematic substance use- fails to yield a significant loading on the Social Role scale. In the one-factor solution, however, the same item loads on the general distress scale, but the loading is quite small. Item 11 -also related to substance abuse- on the Symptom Distress scale has a very small loading, which also improves when the contribution of this item is estimated for the overall symptom distress scale in the one-factor solution. Compared to factor loadings in studies looking at the OQ-45 (for example, Lo Coco et al., 2008) the OQ-30 is more efficient, and concentrates more robust items both for the specifics scales as well as for the overall distress scale. However, the current CFA results are also in line with other research showing that both, the one-factor solution as well as the three-factor solutions yield poor overall fit (Mueller et al., 1998; Beretvas et al., 2003; Lo Coco et al., 2008). Finally, the bi-factor analysis (Simon et al., 2015) yields poor fit indices as well, so that no conclusive evidence can be provided regarding the usefulness of maintaining specific subscales alongside a general scale, or deciding if the OQ-30.2 should be primarily used with attention to the generals core or to the sub-scales.

**Table 2:** Goodness of fit indices for the one-factor, three-factor, and Bi-factor model solution of the OQ-30.2 (*n* = 546).

Model	CFI	TLI	RMSEA
One-factor solution	0.752	0.734	0.074
Three factor solution	0.788	0.770	0.069
Bi-factor model	0.779	0.774	0.067


*CFI, Comparative Fit Index; TLI, Tucker-Lewis Fit Index; RMSEA, Root Mean Square Error of Approximation.*

**Table 3:** Standardized factor loadings for the three-factor and one-factor solution.

Item	Symptom distress	Interpersonal problems	Social role	Symptom distress (overall scale)
1	0.471^∗^			0.420^∗^
2	0.604^∗^			0.543^∗^
4	0.605^∗^			0.523^∗^
5	0.572^∗^			0.534^∗^
6	0.415^∗^			0.471^∗^
7	0.550^∗^			0.496^∗^
8	0.692^∗^			0.633^∗^
10	0.608^∗^			0.545^∗^
11	0.050^∗∗^			0.103^∗∗^
12	0.680^∗^			0.585^∗^
16	0.500^∗^			0.487^∗^
17	0.842^∗^			0.704^∗^
18	0.593^∗^			0.617^∗^
19	0.668^∗^			0.573^∗^
21	0.302^∗^			0.249^∗^
25	0.707^∗^			0.568^∗^
26	0.585^∗^			0.583^∗^
28	0.725^∗^			0.626^∗^
29	0.734^∗^			0.759^∗^
13		0.479^∗^		0.468^∗^
14		0.740^∗^		0.623^∗^
15		0.523^∗^		0.455^∗^
20		0.135^∗^		0.154^∗^
23		0.555^∗^		0.463^∗^
30		0.446^∗^		0.415^∗^
3			0.457^∗^	0.469^∗^
9			0.806^∗^	0.368^∗^
22			0.600^∗^	0.395^∗^
24			0.010	0.099^∗∗^
27			0.825^∗^	0.416^∗^


*^∗^p < 0.01; ^∗∗^p < 0.05.*

### Normative Data

As can be seen in **Table 4**, the OQ normative mean score for the clinical sample (59.21) almost doubles the OQ normative mean score in the non-clinical sample, (29.80) with a similar standard deviation. These normative scores are used in subsequent calculations.

**Table 4:** Normative data for the OQ-30.2.

Sample	*N*	Mean	*SD*
Non-clinical sample	100	29.80	14.00
Clinical sample	546	59.21	16.43


### Cutoff Score

As previously explained, we used Lambert’s original formula to calculate the cutoff score with the data we collected (Lambert et al., 2004). Thus, the cutoff score for the Chilean population is 43.36 points, which is very similar to the score originally found by Lambert et al. (2004) with American samples (*C* = 43.65).

### Reliable Change Index

Reliable change index was also calculated using the formula proposed by Lambert et al. (2004), by calculating the value of the standard error (*S*_E_ = 5.05), and subsequently calculating the error of difference (*S*_Diff_ = 7.14) From these calculations, the RCI within the Chilean population was set at a score of 14. This index can be used to keep track of change in mental health treatment. It should be noted that the calculated cutoff score is almost 4 points higher than that found by Lambert et al. (2004) (RCI = 10.25), which can be explained by the differences in Standard Deviation between Lambert’s clinical samples (*SD* = 13.95) and the present study samples (*SD* = 16.43).

### Reliability

Using the Cronbach alpha coefficient as an expression of the internal consistency of the total scale we calculated that for the clinical sample (*N* = 546), the internal consistency value was α = 0.90. For the control sample (*N* = 100), the internal consistency value was α = 0.88. As can be seen, reliability values for both samples are the within excellent range and are similar to the internal consistency of the original scale.

### Concurrent Validity

The results shown in **Table 5** correspond to the non-clinical sample, as the DAS was only administered to non-clinical participants. Correlations were significant at 0.01 level for each OQ dimension and the OQ-30.2 total score showing strong concurrent validity. It is notable that the strongest correlations occur between the DAS and the items from the OQ-30.2 Symptom Scale, as well as with the total OQ-30.2 score. Conversely, they are lower in the Social Role and Interpersonal Problems scales. These results are expected given that the DAS directly assesses symptoms and not social role or interpersonal relationships. Overall, the correlation coefficients obtained between the DAS and OQ-30.2 are similar to those obtained by Von Bergen and De la Parra, 2002 (0.76 for the non-clinical sample).

**Table 5:** Correlations between OQ-32 scores and DAS scores.

		OQ symptoms distress	OQ social role	OQ interpersonal relationships	Total OQ-30.2
DAS total	Pearson correlation	0.829^∗∗^	0.354^∗∗^	0.673^∗∗^	0.828^∗∗^
	*N*	100	100	100	100


*^∗∗^Significant at p < 0.01 two tailed.*

It is important to note that high correlations may be influenced by the fact that both measures were administered one after the other. This may have caused participants to give similar answers in both questionnaires.

### Responsiveness and Sensitivity to Change

The Responsiveness-Treatment coefficient was computed and yielded a value of 0.52, indicating a moderate responsiveness level. Regarding sensitivity to change, as seen in **Table 6**, there was a significant decreasing slope in the total OQ-30.2 scale. The slope was significantly different from zero, which is expected if change is occurring in the sample as a function of treatment and being captured by the OQ-30.2 over time. More specifically, the slope estimate was -2.456, which goes in the hypothesized direction of overall OQ-30.2 scores decreasing as a function of time during treatment. This slope estimate means that for every session that the patients received individual psychotherapy, the OQ-30.2 scores decrease by 2.456 points up to the fourth session. This is a slope estimate similar to the one produced by Vermeersch et al. (2000) in their sensitivity study, which established that for each session up to the ninth psychotherapy session, the OQ-45 score decreased by 2.2128 points. In Vermeersch et al.’s (2000) study, the slope estimate for the non-clinical sample was significantly lower, at 0.5155 points per measurement instance, up to the ninth assessment.

**Table 6:** Sensitivity to change during psychotherapy.

	*N*	Slope estimate	*SE*	*p*-value
OQ-30.2 Total score	546	-2.456	0.221	0.000


## Discussion

This study assessed the psychometric properties of a Spanish version of the Shortened Outcome Questionnaire (OQ-30.2, Lambert et al., 2004) validated with a sample of 546 patients in an outpatient mental health clinic and 100 non-clinical adults in Chile. As hypothesized, this Spanish OQ-30.2 proved to have similar psychometric properties to the original OQ-30.2 (Lambert et al., 2004). More specifically, this measure has similar normative data, cutoff score, and RCI to the original measure. This Spanish OQ-30.2 is reliable, has concurrent validity with the DAS, and is sensitive to change during psychotherapy, although further studies may be needed to establish sensitivity in a more robust way. As expected, the theoretical three-factor structure was not replicated.

Our results show that the Spanish version of the OQ-30.2 validated in Chile has adequate psychometric properties, with satisfactory internal consistency in both clinical and non-clinical samples and high concurrent validity. In addition, responsiveness was moderate and sensitivity to change was significant, indicating that important changes in the hypothesized direction in patients undergoing psychological treatment can be captured through the measure. The session-to-session change estimate is comparable to estimates reported in the literature so that it can be stated that the OQ-30.2 is both responsive and sensitive to change.

Normative data showed similarities with the normative data obtained by Lambert et al. (2004). The cutoff score for the Chilean population was set at 43.36, which means that scores over 43 will be considered among the dysfunctional population, and vice versa. The RCI dictates that a variation of 14 points or more between sessions is to be considered a clinically significant change.

As seen in **Table 7**, when comparing our results with the results of Von Bergen and De la Parra (2002) and Lambert et al. (2004), it can be observed that the reported values are comparable, and in some cases, our results are even better. Internal consistency measured by the Cronbach Alpha showed similar values (clinical α = 0.90 non-clinical α = 0.88) as those reported by both Lambert (clinical α = 0.93 non-clinical α = 0.93) and Von Bergen and De la Parra (clinical α = 0.91 non-clinical α = 0.91). Sensitivity to change showed a significant paired *t*-value (*r* = 13.55^∗∗∗^), similar to that of Lambert (*r* = 13.75^∗∗∗^) and above Von Bergen and De la Parra (*r* = 6.19^∗∗^). Finally, concurrent Validity showed better results (*r* = 0.83) than what Von Bergen and De la Parra obtained (*r* = 0.76).

**Table 7:** Results of the current study compared to previous studies.

Study	*N*	Internal consistency (Cronbach alpha)	Concurrent validity (DAS correlation)	Sensitivity to change (paired *t*-value)
Errázuriz et al., 2017 (the current study)	Clinical sample = 546 Non-clinical sample = 100	Clinical α = 0.90 Non-clinical α = 0.88	*r* = 0.828^∗∗^	*r =* 13.55^∗∗∗^
Lambert et al. (2004; USA).	Clinical sample = 905 Non-clinical sample = 8,410	Clinical sample α = 0.93 Non-clinical sample α = 0.93	(Not conducted)	*t =* 13.75^∗∗∗^
Von Bergen and De la Parra (2002; Chile)	Clinical sample = 186 Non-clinical sample = 202	Clinical sample α = 0.91 Non-clinical sample α = 0.91	*r* = 0.76^∗∗^	*t =* 6.19^∗∗^


*^∗∗^Significant at the 0.01 level of confidence.*

*^∗∗∗^Significant beyond the 0.0001 level of confidence.*

Regarding the factor structure of the OQ-30.2 this study was not able to provide more empirical support for either the one-factor or the three-factor solution. Further research is probably required to examine the factor structure of the instrument using innovative data-analytic strategies including for example Factor Mixture Modeling, which allows estimating model fit in subpopulations in a given sample.

There are some further limitations to this study. First, concurrent validity was only possible to assess for the non-clinical sample. This is a substantial limitation because of the size of this sample, and it should be addressed in future research. Second, the high correlations between the DAS and the OQ-30.2 raise the question about administration and time. It is possible that respondents tended to give similar answers for both scales, since they answered one after the other and both tend to ask about similar topics. Future research is needed to determine if concurrent validity is being influenced by time of administration. Also, the results of the sensitivity analysis could not be compared to the non-clinical sample, because no longitudinal assessments were conducted for this group. Thus, sensitivity was only established using a slope estimate for the clinical group. Finally, while our total sample (*n* = 646) is larger than the sample used to validate the OQ-45 in Chile (Von Bergen and De la Parra, 2002), it is still a relatively small sample, which may pose constrains to the precision of statistical estimates across analyses.

Overall, given the positive psychometric results reported, and the brief administration time, it is expected that this Spanish version of the OQ-30.2 will be widely used in research, clinical practice, and for the screening of mental health disorders in the aftermath of natural disasters. Additionally, the provided normative data can assist Chilean clinicians in the interpretation of their patients’ psychotherapy outcomes and will contribute to comparable cross-national mental health research. It is a matter of design choices in the setting of future research by Chilean scientists if the shorter, faster and also reliable OQ-30.2 may be a preferable choice when selecting outcome measures.

## Ethics Statement

This study was approved by the Comite Asesor de Bioetica, Fondo Nacional de Desarrollo Cientifico y Tecnologico (FONDECYT), Chile and Comite Asesor de Bioetica, Psicomedica Research Group. Every participant that agreed to be part of this study had to sign two copies of an informed consent, one for themselves and one to be stored by the principal investigator. The consent document details every procedure, the confidentiality of all personal data, and the terms and conditions of participation.

## Author Contributions

PE: Research design, supervised the data collection, statistical analysis, and final manuscript. SO: Data collection, statistical analysis, and wrote the manuscript. AB: Statistical analysis and wrote the manuscript. SG and OS: Data collection and wrote the manuscript.

## Conflict of Interest Statement

The authors declare that the research was conducted in the absence of any commercial or financial relationships that could be construed as a potential conflict of interest.
